# Investing in Interventions to Prevent Opioid Use Disorder in Adolescents and Young Adults: Start-up Costs from NIDA’s HEAL Prevention Initiative

**DOI:** 10.1007/s11121-025-01835-6

**Published:** 2025-10-14

**Authors:** Margaret R. Kuklinski, Brent J. Gibbons, Diana M. Bowser, Kathryn E. McCollister, Rosanna Smart, Laura J. Dunlap, Ella Shenkar, Erin E. Bonar, Tyra Boomer, Mark Campbell, Lynn E. Fiellin, David W. Hutton, Vinod Rao, Lisa Saldana, Katherine Su, Maureen A. Walton, Tansel Yilmazer

**Affiliations:** 1 Social Development Research Group, School of Social Work, University of Washington, Seattle, WA USA; 2 RTI International, Research Triangle Park, Durham, NC USA; 3 Boston College, Connell School of Nursing, Chestnut Hill, Boston, MA USA; 4 Department of Public Health Sciences, University of Miami Miller School of Medicine, Miami, FL USA; 5 RAND, Santa Monica, CA USA; 6 Addiction Center, Department of Psychiatry, University of Michigan, Ann Arbor, MI USA; 7 play2PREVENT Lab at Yale School of Medicine, New Haven, CT USA; 8 Giesel School of Medicine, Dartmouth College, Lebanon, NH USA; 9 Chestnut Health Systems, Lighthouse Institute, Eugene, OR USA; 10 Department of Health Management and Policy, University of Michigan, Ann Arbor, MI USA; 11 Department of Psychiatry, Massachusetts General Hospital, Boston, MA USA; 12 Department of Human Sciences, The Ohio State University, Columbus, OH USA

**Keywords:** Start-up costs, Opioid misuse and opioid use disorder prevention, Older adolescents and young adults

## Abstract

The purpose of this study was to estimate the costs of starting up diverse interventions for preventing opioid misuse in young people aged 15 to 30. Interventions were to be delivered in the context of research trials funded under the National Institutes of Health HEAL (Helping to End Addiction Long-term®) Prevention Initiative. Start-up activities were conducted in systems and settings across the United States from 2019 to 2023. Start-up costs were estimated using activity-based costing from a provider perspective and common methods and data collection procedures across projects (*n* = 8). Descriptive statistics were used to understand the magnitude of and variability in start-up costs, cost drivers, and staff time. Factors explaining variability were identified from project activity descriptions. Start-up activities cost $37,541 on average, and $33,492 at the median (2020 USD). Labor drove costs (89% on average). Training, project management, and partner engagement accounted for 78% of start-up costs on average. There was considerable variability in total and activity costs. Start-up activities provide an essential foundation for successful intervention delivery and sustainability, yet the resources needed and associated costs for this phase of intervention implementation do not receive much attention. We found that interventions faced unique start-up challenges and leverage points, resulting in differences in total cost and activity cost burden. ClinicalTrials.gov Identifier: NCT01088542, NCT04901312, NCT04678960, NCT04617938, NCT04550715, and NCT04135703.

**Supplementary Information:**

The online version contains supplementary material available at 10.1007/s11121-025-01835-6.

## Introduction

The opioid epidemic in the United States remains a major public health crisis. The latest available data show fatal opioid overdoses of approximately 81,000 in 2023, somewhat lower than the roughly 84,000 fatal overdoses reported in 2022 but still a leading cause of unintentional death and still driven largely by fentanyl (National Academies of Sciences, Engineering, and Medicine, 2016). Concerns pertain to all ages, but the opportunity to intervene with youth and young adults to prevent opioid misuse, disorder, and death has become a priority due to rapidly rising morbidity and mortality among this age group. For example, overdose deaths among those aged 15 to 24 quadrupled from 1999 to 2017, driven largely by opioids (Ahrens et al., 2023). Overdose deaths in adolescents aged 12 to 17 rose nearly 30% between 2020 and 2022, mostly due to fentanyl and other synthetics (Baker et al., 2021). In 2022, 1.6% of adolescents aged 12 to 17 and 3.2% of young adults aged 18 to 25 misused opioids; for the same age groups, 1.0% of adolescents and 1.2% of young adults were classified as having an opioid use disorder (Bonar et al., 2020, 2021). The prevalence of alcohol, tobacco, and cannabis use among adolescents and young adults also remains high (e.g., 29.5% of young adults aged 18 to 26 reported binge drinking in the past month) (Bonar et al., 2021; Bowser et al., 2021) and can be an onramp to opioid misuse (Cance et al., 2023; Centers for Disease Control and Prevention National Center for Health Statistics, 2024, Executive Office of the President of the United States, 2022; Dunlap et al., 2023).

Equally concerning are racial and ethnic disparities in overdose morbidity, mortality, access, and engagement in preventive and treatment services among young people. For example, non-Hispanic American Indian or Alaska Native and Hispanic adolescents have significantly higher rates of overdose mortality across all substances compared with other racial and ethnic groups (Cance et al., 2023). Asian, Native Hawaiian or Pacific Islander, and Hispanic adolescents have significantly lower rates of treatment utilization for mental health and substance use disorders compared with White adolescents (Fagan et al., 2019). Racial and ethnic disparities in opioid morbidity, mortality, and service access are also found across young adulthood (Florence et al., 2021; Friedman et al., 2022; Goldstein et al., 2023).

The opioid crisis has mobilized efforts to prevent and treat opioid use disorder across many relevant parties and systems, including clinicians, behavioral health providers, criminal justice, education, and third-party payers. In 2018, the National Institutes of Health (NIH) developed the Helping End Addiction Long-term® (HEAL) Prevention Initiative to address the overdose crisis (NIH Health Initiative, 2023; Hudgins et al., 2019; National Institute of Health, n.d.). Because of increasing concerns about opioid misuse in older adolescents and young adults, alongside an absence of evidence-based opioid misuse prevention interventions (Executive Office of the President of the United States, 2022), NIH established the HEAL Prevention Cooperative (HPC), which supports 10 research projects and one coordinating center and is administered by the National Institute on Drug Abuse (NIDA). Projects are developing, implementing, and testing a variety of interventions to prevent opioid use and misuse among 15- to 30-year-olds (Jolles et al., 2024). Although they share a common goal, the 10 interventions span the prevention continuum (i.e., from universal to indicated interventions) (Kaplan, 1994) and are being offered in a variety of settings (e.g., healthcare, community, school, juvenile justice). They also address populations that are underserved, understudied, and at higher risk for misuse, disorder, and morbidity (Table1). Comprehensive study summaries are published in several protocol papers and on the HPC website (Komro et al., 2023; Larochelle et al., 2021; Lee et al., 2025; Lu et al., 2021; Luo et al., 2021; Miech et al., 2023).

**Table 1:** HEAL prevention interventions, United States, 2019–2023

Project lead^a^	Intervention^b,c^	Target population	Setting	Months to start-up	Intervention sites	Planned intervention enrollment
Chestnut Health Systems	PRE-FAIR: integrated preventive intervention, including evidence-based substance use and mental health treatment, parent skills training, and intensive case management	Young parents ages 16–30 involved with or at risk for involvement with child welfare and self-sufficiency services	Community-based clinics with social services referrals	8	2	120
Harvard MGH	BHTx: implementation of patient-reported outcomes measures into clinical care/electronic health record to screen for OUD/SUDs and other psychopathology	Youth ages 16–30 receiving treatment for mental health disorder or comorbid mental health and non-opioid substance use disorder	Hospital, behavioral health, and substance use disorder clinics	24	1	6891
OSU	HOME: provision of housing as well as opioid and related risk-prevention services	Homeless youth ages 18–24 who do not have OUD	Drop-in centers, shelters, broader community	5	1	120
RAND/UCLA	TACUNA: group-based motivational interviewing to address opioid, alcohol, and cannabis use through discussion of social networks and engagement in traditional practices, combined with monthly community wellness circles	English-speaking American Indian/Alaska Native young adults ages 18–25 living in urban areas who do not have opioid dependence at study baseline	Community; virtual	15	1	223
SCRI/UW	POST: assertive community care/assertive continuing care-based OUD prevention interventions of various intensity levels; more intensive arm also includes trauma-focused intervention	Youth ages 15–25 re-entering the community after justice involvement	Juvenile legal	20	10	215
TCU	LeSA: adapted evidence-based prevention intervention called Trust-Based Relational Intervention that includes group sessions while youth are in custody and coaching visits post-release	Youth ages 15–25 transitioning to the community after detainment in a secure treatment or correctional facility	Juvenile legal	44	13	172
UM	Michigan: ED-based, video-delivered, single session with a health coach and post-ED web-based messaging with health coaches in a portal-like platform for 30 days using motivational interviewing strategies	Youth ages 16–30 who present to the ED and report past-12-month opioid misuse or report past-12-month prescription opioid use with accompanying risk factor	Hospital EDs	17	2	960
Yale/Dartmouth	VidGame: video game intervention to prevent initiation of opioid misuse delivered in schools (some with school-based health centers)	Youth ages 16–19 who are preferably enrolled in a school-based health center, report not having engaged in prior opioid misuse, and have at least one of three risk factors	High school and school-based health center	25	15	266

*HEAL* Helping to End Addiction Long-term®

^a^*MGH* Massachusetts General Hospital, *OSU* The Ohio State University, *SCRI* Seattle Children’s Research Institute, *TCU* Texas Christian University, *UCLA* University of California at Los Angeles, *UM* University of Michigan, *UW* University of Washington

^b^*BHTx* Behavioral Health Treatment, *HOME* Housing Opportunities, Motivation, and Engagement, *LeSA* Leveraging Safe Adults, *POST *Positive Outcomes through Supported Transitions, *PRE-FAIR* Families Actively Improving Relationships for Prevention. Families Actively Improving Relationships (FAIR©™) is trademarked and copyrighted in accordance to United States Law by Oregon Social Learning Center (OSLC), *TACUNA* Traditions And Connections for Urban Native Americans

^c ^*ED* Emergency department, *OUD* Opioid use disorder, *SUD* Substance use disorder

In addition to evaluating effectiveness, projects were required to conduct economic analyses, including assessing start-up costs (to inform resources needed to get an intervention up and running), cost effectiveness (to inform the relative value of different interventions), and budget impacts (to inform the budget needed and potential outcomes when considering scaling up an intervention) (National Institute of Health, n.d.). Understanding startup is especially important because capacity building and other preparations lay an essential foundation for impact, yet these costs are generally not reimbursable through the same funding streams as direct service. Initiating a substance use prevention program might be quite different from starting a substance use treatment program. For example, while treatment generally occurs in some type of healthcare setting (e.g., hospitals, specialty clinics) in which services are billable, prevention can occur in a variety of settings (e.g., schools, juvenile legal facilities, community-based agencies) in which services generally are not reimbursable through insurance mechanisms. In addition, because prevention generally is underutilized as a strategy for combatting the opioid crisis and substance misuse more broadly, new initiatives could involve significant effort to bring new partners together to establish new working relationships, train staff in new techniques, develop new workflows, and the like. Unlike treatment interventions, which may require specialty staff with advanced skills and training, preventive interventions often can be delivered by staff without advanced credentials, which might reduce some start-up challenges, like the need to hire new staff. These differences suggest that starting up prevention initiatives may lead to a variety of costs, though the magnitude and distribution of these costs is not well understood.

Despite its importance, start-up costs have typically not received as much attention as service delivery costs in economic evaluation studies (Montoya et al., 2024; Moullin et al., 2019). The HPC offers a unique opportunity to assist funders and system leaders considering prevention initiatives in gaining insight into start-up costs by examining a set of diverse prevention interventions—deployed in real-world settings, often outside of the traditional healthcare sector—that are united by the common goal of preventing opioid misuse and disorder in adolescents and young adults. Several projects specifically focused on populations and settings in which young people were at a higher risk of opioid misuse and disorder. This study aims to understand the magnitude of and variability in start-up costs across the set of projects, including use of specific resources (e.g., labor, materials, and supplies) and the types of start-up activities (e.g., project management, training, policy, and procedure development) in which project teams engaged. A primary objective was to identify the specific resources and activities that drove start-up costs. We also wanted to identify factors that helped explain variability in start-up costs and cost drivers. We expect study findings to generate greater understanding about the economics of implementing opioid/substance use disorder prevention interventions in the United States, specifically in relation to the foundational start-up phase.

## Methods

### Sample and Procedures

The HPC convened the Health Economics Workgroup (HEWG), a collaboration between economists from the research projects and the coordinating center, to develop and execute standardized approaches to economic evaluation that would enhance the validity and meaning of analyses using data from multiple projects. Eight of 10 HPC projects participated in the cross-project start-up cost analysis (Table 1). Health economists from each project and the coordinating center convened in regular HEWG meetings from the beginning of the cooperative to develop a shared approach to start-up cost data collection and cross-project analysis. Rigorous discussion centered on a methodology for estimating start-up costs, how to collect relevant data, the collection period, and how to apply wages as key drivers of costs in a systematic and standardized way. HEWG members shared their expertise, reviewed relevant research literature (National Institute of Health, n.d., O*NET, 2024; Pandika et al., 2022), and consulted with the HPC’s steering committee (composed of project principal investigators and overall HPC leadership). After several months of deliberation during the project’s first year, the HEWG reached consensus on using activity-based costing (Pendergrass Boomer et al., 2023) from a provider perspective (i.e., the perspective of those delivering the intervention), focused on six distinct cost categories expected to adequately capture activities undertaken during project start-up: meetings to engage key partners outside of the core research project (i.e., partner engagement); initial staff training for intervention implementation; initial staff hiring and acquisition of necessary equipment and supplies (e.g., purchase of licensed software); development and revisions to policies and procedures to accommodate organizational workflow and processes (e.g., producing a manual summarizing intervention workflows); project management (e.g., weekly team meetings); and an “other” category that included costs such as promotional and marketing materials. To aid in interpreting cost estimates, each project described sub-activities within each broader activity category, such as the type of training delivered and the staff types involved. Because research staff were often present at multipurpose meetings but not substantively involved in start-up activities, we did not include their time in start-up cost estimates. Only time engaged in non-research intervention start-up activities was counted. For each project, the time horizon for start-up costs was from when intervention preparations began at a site to when the first participant enrolled in intervention services.

The HEWG standardized unit costs for labor across projects in order to remove the influence of local and regional wage variation on cost differences across projects. It did this by applying national wage estimates (2020 USD) from the Occupational Information Network (O*NET) (Reddy et al., 2021) to labor hours data collected by projects. To determine which O*NET job classifications to use, economists worked with their project teams to identify the staff titles and occupations for their own projects. The HEWG then mapped this information to O*NET occupations and national wage estimates. If a job title did not exactly match the O*NET list, project staff chose the most relevant title based on tasks performed (e.g., a case manager was matched to the O*NET occupation “mental health and substance use social workers”). The HEWG also applied a 30.2% rate for fringe benefits (e.g., employer insurance costs, paid leave, Social Security, Medicare, unemployment insurance), which represents an average of private and civilian rates (Ridenour et al., 2023).

### Data Collection and Start-up Cost Estimation

To facilitate consistent prospective data collection across projects, the HEWG developed an Excel-based data collection instrument that was tailored by projects to capture labor and non-labor resource use and costs for each of the six start-up activities. HEWG members worked with staff on their respective projects to develop a project-specific prospective data collection plan executed by the project coordinator or another knowledgeable team member. The intent was to record labor time regularly, as close as possible to the time it was incurred, without being overly burdensome to busy staff, in order to reduce recall bias and inaccuracies (e.g., weekly updates to the template by project coordinators, weekly prompts from project coordinators to start-up staff to enter hours by activity into a REDCap or Qualtrics database). In this way, data collection was harmonized (identical data collection categories) but not standardized (some variation in measurement).

Following activity-based costing methodology, each project collected data on the quantity and unit price of resources (contracted services; travel; and materials, supplies, and equipment) used and time spent by staff involved in startup across the six mutually exclusive start-up activities. Within each activity, the cost of each resource was then estimated as the resource quantity multiplied by its unit price. Resource costs were summed to yield an activity cost. Activity costs were summed to yield total start-up costs. To estimate labor costs within each activity, time estimates for each role were multiplied by the corresponding O*NET wage estimate. Labor costs were summed for each activity and across activities to generate a total labor cost. Each project calculated the proportion of costs attributable to labor in total and by activity by dividing labor cost by total cost or activity cost, respectively.

To facilitate understanding of staff required for startup, each project classified staff roles (and associated time in start-up activities) into three broader staffing categories: clinical intervention and delivery, partners/facilitators, and management and administration. Clinical intervention and delivery included staff who implemented the intervention and their supervisors. Partners/facilitators included staff whose support and buy-in indirectly facilitated intervention implementation (e.g., key leaders not involved in day-to-day operation of the intervention). Management and administration included all staff involved in the start-up phase who were not clinical intervention and delivery staff or partners/facilitators. Projects reported total time spent in start-up activities in total and for each of these staff categories.

### Analysis Strategy

We described each project in terms of intervention, population served, delivery setting and number of sites, and expected enrollment in the research trial. We used descriptive statistics (e.g., mean, median, standard deviation) to understand start-up costs (2020 USD) and their variability in total, by resource (labor, non-labor), and by activity, and also to understand start-up hours in total and by staff type (management, clinicians, partners/facilitators). We also estimated start-up costs per enrollee expected in the research trials and per intervention site to normalize costs to some degree. To see if start-up costs varied in relation to start-up length, number of study sites, and planned enrollment, we examined univariate correlations. To understand the main activity and resource cost drivers across the set of interventions, we first estimated the proportion of costs by activity and resource for each project and then used descriptive statistics to understand overall patterns and their variability. To better understand start-up costs for labor, which we expected to be a key cost driver, we examined the mean and median time spent in start-up activities across the three staff categories. We used intervention descriptions, including sub-activities within each broader activity category, to identify factors explaining variability in costs. These factors were reviewed and refined by HEWG members and principal investigators.

## Results

### Interventions

Interventions had a common goal of preventing opioid misuse and/or opioid use disorder among adolescents and young adults, but they differed in type and intensity, populations served, delivery settings and number of sites, and expected enrollment (Table 1). Across all projects, start-up activities were conducted between 2019 and 2023; for individual projects, they varied from 5 to 43.5 months and were influenced by several factors including the research design, intervention complexity, and implementation setting.

### Start-up Costs and Cost Drivers

On average, start-up costs were $37,541 in total, $18,278 per site, and $209 per planned enrollee (Table 2). Median costs were lower ($33,492 total, $13,461 per site, $128 per enrollee) because cost distributions were skewed to the right.

**Table 2:** Cost of starting up HEAL prevention interventions, United States, 2019-2023 (2020 USD)

Statistical measure	Start-up sites	Planned enrollees	Start-up costs^a^			Share by resource^b^		Share by activity^c^					
			Total	Per site	Per planned enrollee	Labor	Non-labor	Train-ing	Project manage-ment	Partner engage-ment	Policy and procedure develop-ment	Acqui-sition and hiring	Other
Median	2	219	$33,492	$13,461	$128	95%	6%	26%	13%	15%	8%	1%	0%
Mean	5	697	$37,541	$18,278	$209	89%	11%	32%	23%	22%	13%	7%	2%
Standard deviation	5	1166	$30,675	$18,277	$283	14%	14%	23%	23%	28%	15%	13%	7%
Minimum	1	120	$4,194	$419	$2	62%	0%	4%	0%	3%	0%	0%	0%
Maximum	13	6891	$104,128	$52,064	$868	100%	38%	80%	62%	87%	38%	37%	19%

HEAL = Helping to End Addiction Long-term®

^a^ Start-up costs are across eight projects funded under the HEAL Prevention Initiative

^b^ Values reflect the proportion of total start-up costs attributed to labor and non-labor, respectively. Minimum values of zero arose because two projects did not report any non-labor costs

^c^ Values reflect the proportion of total start-up costs attributed to each of the six start-up activities. Minimum values of zero arose because one project did not report project management costs, three projects did not report policy and procedure development costs, three projects relied on existing staff and thus did not report acquisition and hiring costs, and six projects did not report costs outside of the other five activities (also reflected in the zero median value for other costs)

From a resource perspective (Table 2), labor was the main cost driver, accounting for 89% of start-up costs on average, and even more at the median. It also dominated costs for each activity category (e.g., 77% of acquisition and hiring, 92% of partner engagement; analyses not shown) except for “other,” in which materials and supplies were the main source of cost. Labor time (see Table S1, available online) averaged 886 h in total (462 per site, 5 per planned enrollee) but was lower at the median (571 in total, 247 per site, 2 per planned enrollee). Labor consisted primarily of management (46% of start-up hours, on average) and clinical (47%) staff. Site partners and facilitators comprised less than 10% of start-up hours on average.

Training (32% of start-up costs, on average), project management (23%), and partner engagement (22%) were the largest share of costs (Table 2), together accounting for 77%, on average. Acquisition and hiring, policy and procedure development, and other activities comprised far smaller shares.

### Variability in Start-up Costs and Cost Drivers

**Fig. 1:**
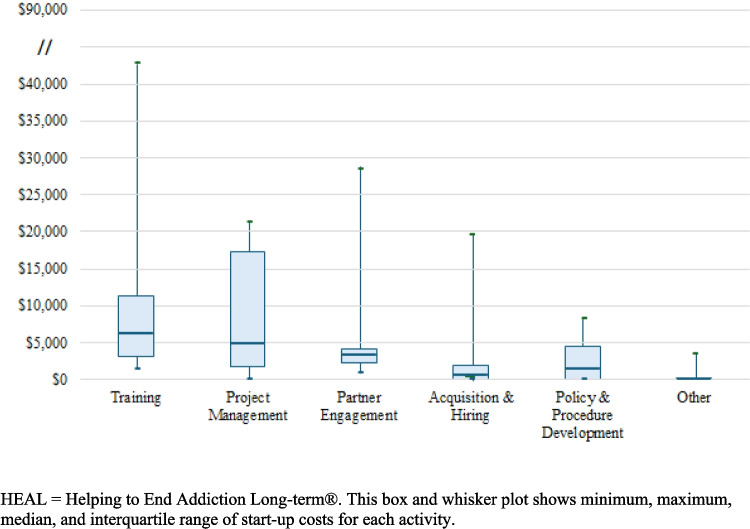
Cost of start up activities, HEAL prevention interventions, United States, 2019–2023 (2020 USD)

Start-up costs varied substantially (Fig. 1 and Table 2; range: $4,194 to $104,128 in total; $419 to $52,064 per site; $2 to $868 per planned enrollee). Variability was not explained by the length of startup (*r* = −.41, *p* =.31), the number of study sites (*r* = −.28, *p* =.50), or planned enrollment (*r* = −.25, *p* =.54). It was also not explained by differences in labor type used or associated labor unit costs, which drove costs for all projects, nor by delivery setting. Rather, differences in level of resource intensity for a given activity explained variation in start-up costs (Table 2; Fig. 1). Patterns were complex, such that projects typically incurred higher costs or greater resource needs in relation to one or two specific activities (e.g., training, or training and project management), rather than consistently high or low costs across all activities (Table 3). For example, *training costs* were higher and/or a larger share of the total cost when interventions were complex or multicomponent, new staff needed to be trained, or interventions were to be delivered 1:1 with populations at higher risk. They were lower when existing trained staff could be deployed to the intervention or when interventions were delivered digitally. *Project management costs* were higher and/or a larger share for interventions that involved multiple community partners (requiring resources for establishing partnerships and gaining buy-in) or were to be delivered in healthcare systems (given the complexity of interfacing with existing operations and workflows). *Partner engagement costs* were higher when sites had to establish relationships with implementation or delivery sites, especially criminal–legal, which has in place protections for youth in confinement, or had a large number of community partners to navigate. They were generally lower among projects that had well-established relationships with implementation or delivery settings. *Acquisition and hiring costs* were generally low in magnitude and share unless several new staff needed to be hired and/or equipment purchased. *Policy and procedure development costs* were generally higher for projects requiring more partner engagement or for complex 1:1 interventions delivered in criminal–legal settings. It was rare for projects to incur start-up costs outside of these five activities. Table 3 includes some examples showing how resource intensity across multiple activities could lead to lower or higher start-up costs. They are not reflective of specific projects but point to the collective influence of multiple start-up activities on total start-up costs.

**Table 3:** Factors associated with lower or higher start-up costs

Lower start-up costs	Start-up activity	Higher start-up costs
• Uses existing trained workforce • Few trainees (e.g., group-format intervention) • Digitally delivered intervention requiring few staff to support	Training	• Multicomponent intervention • Many trainees (e.g., 1:1 intervention, potentially dispersed across many different sites) • Planning for scale: Need for intensive training (e.g., for entirely new service model requiring new skillsets)
• Fewer partners	Project management	• Multiple partners • Healthcare setting
• Established relationships with delivery setting • Fewer partners	Partner engagement	• New delivery setting, especially protected criminal legal setting • Multiple partners
• Leverages existing staff • No or low materials, supplies, and equipment requirements	Hiring and acquisition	• Many new staff need to be hired • Equipment needs to be purchased or other capital investments need to be made
• Able to use existing workflows	Policy and procedure development	• Multiple partners needing coordination • Complex interventions requiring clear workflow specifications
• A digitally delivered intervention offered at sites with pre-existing prevention infrastructure • A group-based intervention using trained staff offered in a setting with established relationships with project leads	*Hypothetical examples cutting across activities*	• A multicomponent intervention offered 1:1 among youth at higher risk for OUD and needing significant training and well-specified policies and procedures • An intervention offered 1:1 necessitating many new hires and capital investments and integration into complex existing system workflows

*HEAL* Helping to End Addiction Long-term®, *OUD* Opioid use disorder

## Discussion

Intervention startup has rarely been a primary focus of prevention cost studies (Montoya et al., 2024; Moullin et al., 2019), yet by ensuring readiness for intervention delivery by well-trained staff, with adequate supplies and systems support for intervention, start-up activities provide an essential foundation for potential efficacy and sustainability. Across the eight HPC projects studied here, start-up costs were roughly$34,000 at the median and $128 per planned enrollee (2020 USD). Most of these costs were attributable to labor, which at the median totaled 600 h (equivalent to 0.29 FTE for 1 year spread across several positions), primarily among management and clinical staff. Start-up cost drivers varied, but generally, training, project management, and partner engagement accounted for the majority of costs.

Costs were estimated in the context of research trials designed for impact in a variety of populations and settings, including those serving older adolescents and young adults at higher risk for opioid misuse and/or opioid use disorder and with access to funding for start-up activities. In non-research settings, mechanisms for financing prevention startup are rarer. Preventive interventions receive little funding relative to treatment (Singh et al., 2019; Spacirova et al., 2020), accounting for only 5% of total healthcare spending and less than 10% of federal drug control spending (Substance Abuse and Mental Health Services Administration, 2021). Monies typically are reserved for intervention delivery, rather than pre-intervention preparation and capacity building. This study’s findings suggest that rigorous preparation need not be high in cost, even for interventions designed for complex systems and for populations at higher risk, but these costs also are not incidental (i.e., six of eight projects cost $17,000 to $52,000 [2020 USD]). These investments, which fell between $2 and $900 per participant, are far below the annual cost of a single case of opioid use disorder, estimated at $221,219 (2017 USD) in 2017, and even more so the cost per case for fatal opioid overdose, estimated at $11,548,000 (2017 USD) in 2017 (Substance Abuse and Mental Health Services Administration, 2023, 2024). The high costs of disorder and overdose give reason to believe that effective preventive interventions could yield substantial cost savings, even after accounting for startup. The HPC’s planned prevention cost-effectiveness and budget impact studies are needed to provide empirical evidence supporting this claim (National Institute of Health, n.d.).

Potential adopters working in clinical and nonclinical settings need access to funding and mechanisms that speed translation from prevention demonstration project to routine service delivery. This includes ensuring that start-up activities are reimbursable or covered in funding mechanisms in order to more broadly incentivize the delivery of preventive services. If such mechanisms were more readily available, the cost to start up new interventions would likely decrease over time because existing prevention infrastructure could be harnessed for new initiatives. Without such mechanisms, systems with lean budgets may find it difficult to justify and fund preventive activities, especially if cost savings accrue outside these systems. Costs of illness studies indicate substantial gains in productivity and lower health care, substance use treatment, and criminal legal costs when opioid use disorder is averted (Substance Abuse and Mental Health Services Administration, 2023). However, systems other than these may invest in the preventive interventions that lead to broad cost savings. Sustainable funding for prevention would make the decision to invest in prevention, regardless of where savings accrue, much easier.

Projects varied in whether they were building prevention capacity for long-term delivery or providing proof of concept through efficacy trials. Regardless, examining costs per planned trial enrollee suggests that potential for economies of scale might be realized as an intervention’s reach increases. All but one project cost less than $300 per planned enrollee, equating to a few hours of staff time (Table S1). If interventions and infrastructure were available to more young people after research trials were over (as some projects intended to do), costs per enrollee would continue to decline for many interventions (until utilization reached capacity), increasing the return on investment in start-up activities.

These findings advance understanding and estimation of start-up costs. Results showed that six activities (training, project management, partner engagement, acquisition and hiring, policy and procedure development, and a rarely used “other” category) adequately captured start-up preparations across a variety of interventions and delivery settings; these activities can serve as a template for future start-up cost analyses undertaken for a variety of projects and conducted in a variety of settings. The project developed a process and tools for standardizing and harmonizing data collection and cost estimates across projects and the six activity cost categories that could be used in future studies.

For most of the eight projects, one or two activities drove costs (Table 2; Fig. 1). Although our sample is not large enough to rigorously examine costs by setting type, it did include two projects in the healthcare system and two in the juvenile legal system. The healthcare-based projects differed greatly in start-up costs due to differences in the complexity of partner engagement, training, and project management. The two projects in the juvenile legal system had similar costs, but activity cost drivers differed. A potential lesson for future adopters of new prevention initiatives is to anticipate some relatively challenging aspects of the preparation phase irrespective of the system or setting involved. These challenges and associated start-up costs are likely to reflect the nature of the intervention (i.e., labor-intensive 1:1 intervention compared with digital delivery), different points of leverage (i.e., creating new compared with using existing capacity and infrastructure), whether relationships already exist between project and system leaders and managers, and project leadership decisions about where investments are most needed to achieve objectives of the intervention and setting.

The types of costs incurred during startup and the differences in cost drivers across the projects studied are consistent with frameworks supporting dissemination and implementation. For example, both EPIS (Exploration, Preparation, Implementation, and Sustainment) (U.S. Bureau of Labor Statistics, 2023) and PRISM (Practical, Robust, Implementation and Sustainability Model) (Volkow et al., 2021) point to the need for support and buy-in from key leaders as new initiatives are being implemented. It is understandable that for projects deployed in new settings, partner engagement costs, policy and procedure development costs, or both would be relatively high. Both EPIS and PRISM also suggest that the availability and adequate preparation of staff is essential to the success of new initiatives. From that lens, it is not surprising that training costs were often significant drivers of start-up costs. Although this study’s start-up cost examination was not specifically guided by existing implementation science frameworks, future efforts to examine prevention start-up costs may benefit from doing so.

### Limitations

This study had some limitations. Eight of 10 projects funded under the HPC were included. Although it is rare to be able to compare start-up costs across multiple projects, a larger sample would be desirable. Interventions are being delivered in the context of research trials funded by NIH, with startup set at 1 or 2 years by the funding mechanism. Startup outside of the trial context might take more or less time. In addition, some activities for some projects might have been more intensive (e.g., more staff were trained) within the research setting, while others might be more intensive in some settings outside the trial context (e.g., if new staff need to be hired). Because the COVID-19 pandemic began during the start-up period, many activities shifted from in-person to virtual, potentially saving costs for travel to in-person activities but increasing acquisition costs because hiring new staff was difficult. Resource and cost measurement was harmonized but not completely standardized. Differences in data collection procedures and other sources of method variance may explain some differences in intervention cost estimates. Overall, limitations suggest some imprecision in estimates and point to some differences in start-up costs to be expected outside the context of a research trial. However, they do not detract from this project’s findings about start-up cost drivers and factors explaining variability in these costs.

## Public Health Implications

Opioid misuse prevention is a critical part of an overall strategy to reduce the opioid crisis and related harms, particularly in the next generation of adults, but effective interventions for older adolescents and young adults, including those at higher risk, are lacking. To ameliorate this situation, NIH, with lead funding from NIDA, has funded a set of projects and trials aiming to establish effective, feasible, and sustainable preventive interventions for this age group. Interventions cost less than $40,000 on average to start up in a range of real-world settings. Study findings illuminate the kinds of challenges that can lead to higher start-up costs and also the factors that can reduce start-up costs. They indicate the nature of the new initiative, and the extent to which it can capitalize on existing staff, workflows, partnerships, and the like, and influence total start-up costs and the activities that drive cost. Patterns identified should be useful to other potential funders and system leaders considering adopting new prevention initiatives. Additional research establishing intervention cost-effectiveness and budgetary impact when implemented within a variety of systems and agencies, along with policy reforms that increase sustainable financing for prevention, would help ensure these interventions are feasible and sustainable and reach those who could benefit from prevention and intervention efforts focused on opioid misuse.

## Supplementary Information

Below is the link to the electronic supplementary material.

Supplementary Material 1 (DOCX 24.9 KB)

Supplementary Material 2 (XLSX 22.1 KB)
