# Supplementary material for: Investing in Interventions to Prevent Opioid Use Disorder in Adolescents and Young Adults: Start-up Costs from NIDA’s HEAL Prevention Initiative
Source: Prev Sci. 2025 Oct 14;26(7):1045–55. doi: 10.1007/s11121-025-01835-6 (PMC12627165; doi:10.1007/s11121-025-01835-6)
Supplement: Supplementary file 1 — Supplementary Material 1 (DOCX 24.9 KB) [file 11121_2025_1835_MOESM1_ESM.docx]

| Table S1. Start-up Labor Time, HEAL Prevention Interventions, United States, 2019-2023 | | | | | | | |
| --- | --- | --- | --- | --- | --- | --- | --- |
|  | Start-up Hours | | |  | Share by Staff Type | | |
| Project Lead | Total | Per Site | Per Planned Enrollee |  | Management | Clinical | Partners & Facilitators |
| Median | 571 | 247 | 2 |  | 42% | 43% | 3% |
| Mean | 886 | 462 | 5 |  | 46% | 47% | 7% |
| Standard Deviation | 881 | 522 | 8 |  | 21% | 18% | 9% |
| Minimum | 118 | 12 | 0 |  | 17% | 16% | 0% |
| Maximum | 2826 | 1413 | 24 |  | 84% | 68% | 25% |
